# Supplementary material for: Erythroferrone Modulates Osteoblast-Osteoclast Crosstalk During Bone Remodeling
Source: bioRxiv. 2026 May 8:2026.05.05.722997. Preprint. [Version 1] doi: 10.64898/2026.05.05.722997 (PMC13174593; doi:10.64898/2026.05.05.722997)
Supplement: Supplement 1 [file media-1.pdf]

Supporting Information for

Erythroferrone Modulates Osteoblast-Osteoclast Crosstalk During Bone Remodeling

Pinanong Na-Phatthalung[1,2], Gabrielle van Caloen[1,2], Marina Planoutene[1], Emily Tai[1], Anisa Gumerova[2,3], Georgii Pevnev[2,3], Jay Cao[4], Ronit Witztum[1,3], Eva Ingber[1], Leon Kautz[5], Farhath Sultana[2,3], Funda Korkmaz[2,3], Maayan Levy[1], Tony Yuen[2,3], Mone Zaidi[2,3*], Yelena Z. Ginzburg[1,2*]

*Address Correspondence to:

Yelena Z. Ginzburg, MD

The Tisch Cancer Institute Division of Hematology and Medical Oncology

Icahn School of Medicine at Mount Sinai

1 Gustave L. Levy Place, Box 1079 New York, NY 10029. Tel (212) 241-0579.

Email: yelena.ginzburg@mssm.edu

This PDF file includes:

Figures S1 to S5

Tables S1 and S2

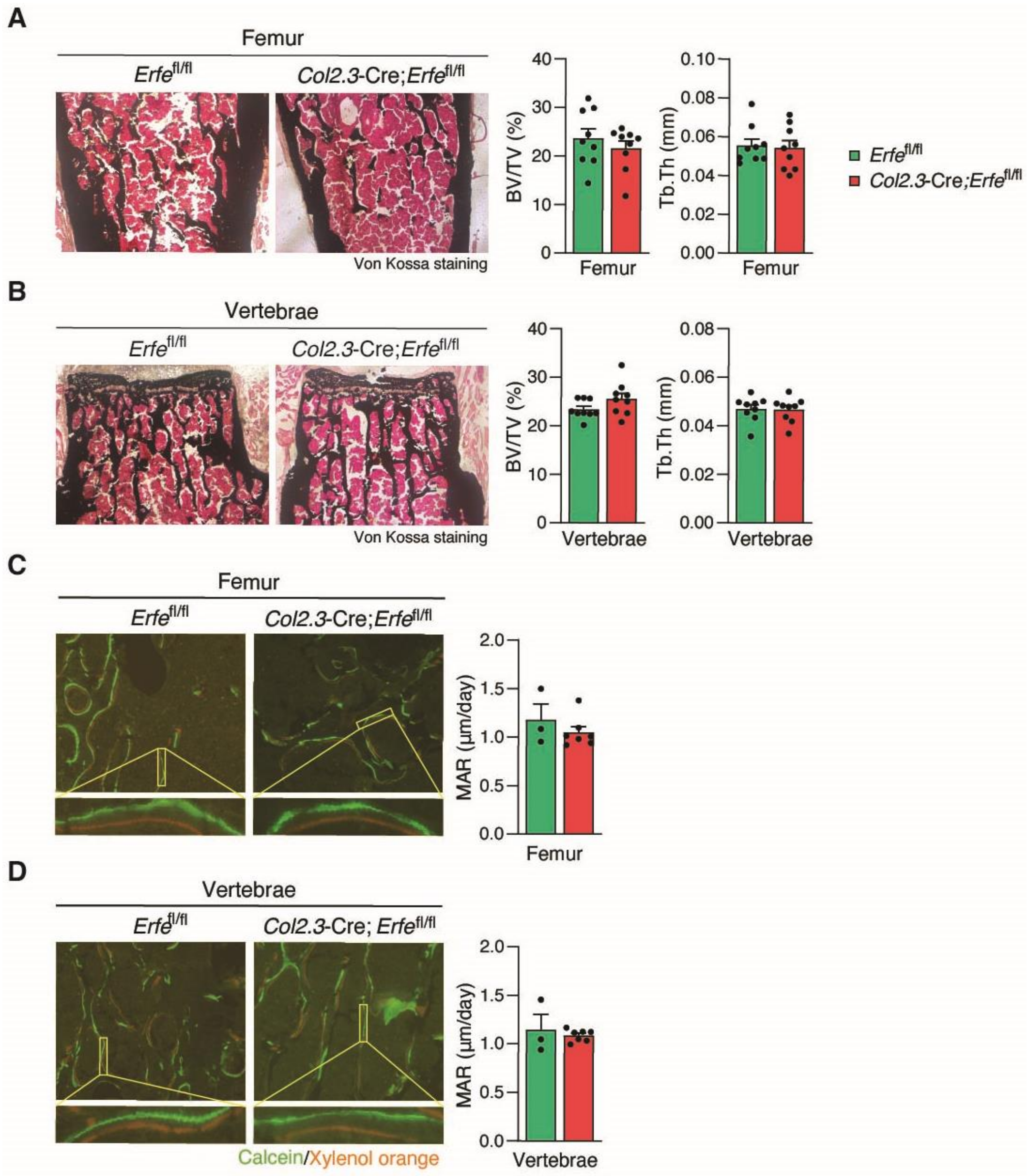

<u>Figure S1</u>: **Unchanged Calcium Deposits and Appositional Bone Growth in Osteoblast-Selective *Erfe* Knockout Mice.** (**A**-**B**) Von Kossa staining images of the femur and lumbar vertebrae and its histomorphometric measurements for bone volume fraction (BV/TV) and trabecular thickness (Tb.Th) from 14-week-old *Erfe*$^{fl/fl}$ and *Col2.3*-Cre;*Erfe*$^{fl/fl}$ male mice (N = 9). (**C**-**D**) Fluorescent images of mineral apposition rate (MAR) determined by the distance between the lines of mineral deposition from calcein green and xylenol orange injections (N = 3-5). Data are presented as mean ± SEM.

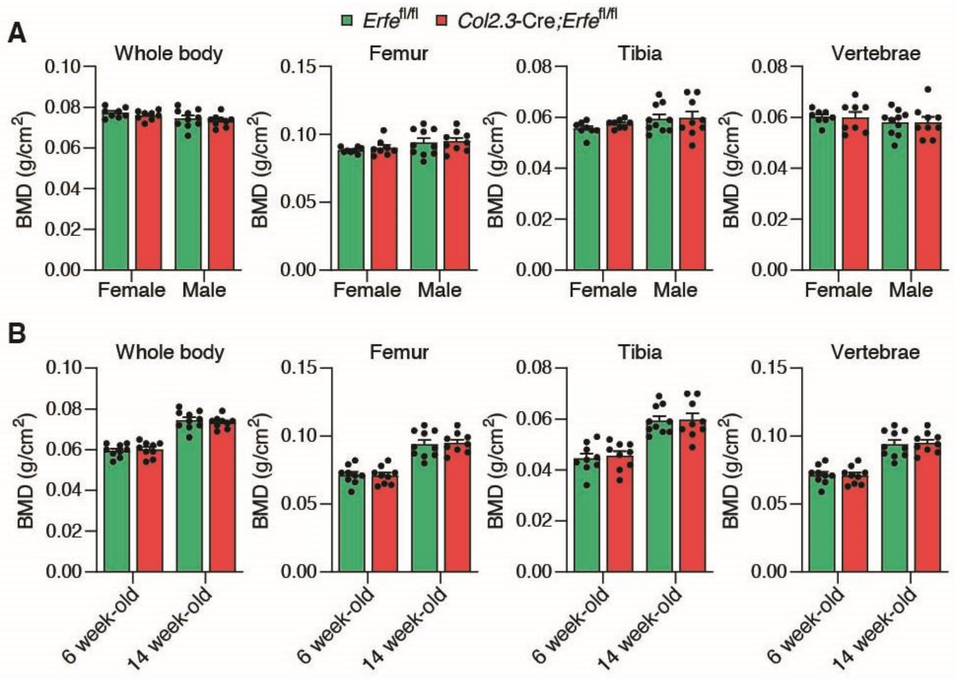

<u>Figure S2</u>: Osteoblast-Selective *Erfe* Knockout Mice Do Not Exhibit Age- and Gender-Specific Differences in Bone Characteristics. (**A**) Comparison of bone mineral density (BMD) in the whole body, femur, tibia, and lumbar vertebrae (L4-L6) measured by DXA scan between 14-week-old *Erfe*$^{fl/fl}$ and Col2.3-Cre;*Erfe*$^{fl/fl}$ male and female mice (N = 9). (**B**) Comparison of BMD between 6-week-old and 14-week-old *Erfe*$^{fl/fl}$ and Col2.3-Cre;*Erfe*$^{fl/fl}$ male mice (N = 9). Data are presented as mean ± SEM.

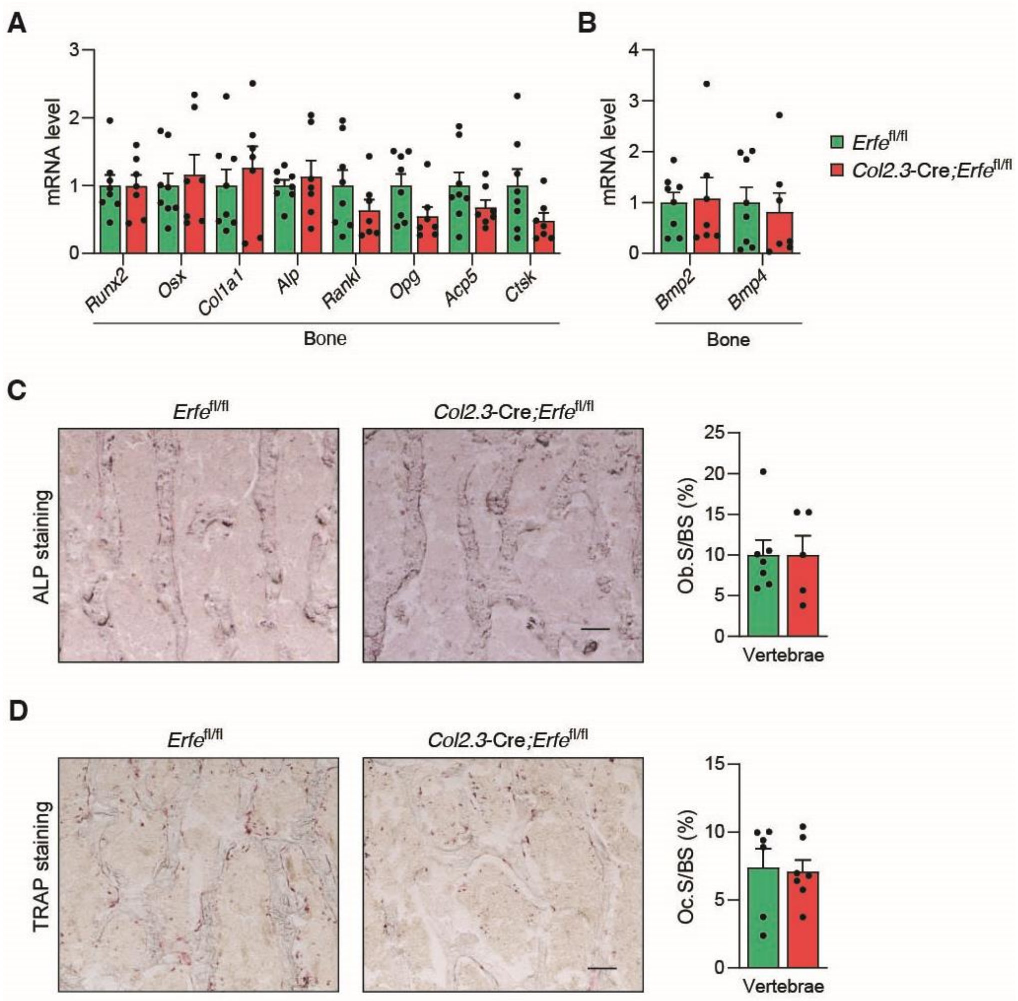

<u>Figure S3</u>: *Erfe* Loss in Osteoblasts Does Not Affect Gene Expression and Cellularity of Osteoblasts and Osteoclasts in the Bone *In Vivo*. (**A**) mRNA levels of osteoblast and osteoclast biomarker genes; *Runx2*, *Osx*, *Col1a1*, *Alp*, *Rankl*, *Opg*, *Acp5* and *Ctsk* in the bone of 14-week-old *Erfe*$^{fl/fl}$ and Col2.3-Cre;*Erfe*$^{fl/fl}$ mice (*N* = 3-4). (**B**) *Bmp2* and *Bmp4* mRNA levels in the bone of *Erfe*$^{fl/fl}$ and *Col2.3*-Cre;*Erfe*$^{fl/fl}$ mice (*N* = 3-4). (**C**-**D**) Representative images of ALP and TRAP staining and percentage of osteoblast and osteoclast surface determined by bone morphometric analysis of the vertebrae from 14-week-old *Erfe*$^{fl/fl}$ and *Col2.3*-Cre;*Erfe*$^{fl/fl}$ mice (*N* = 5-7). Scale bar 0.1 mm. Data are presented as mean ± SEM.

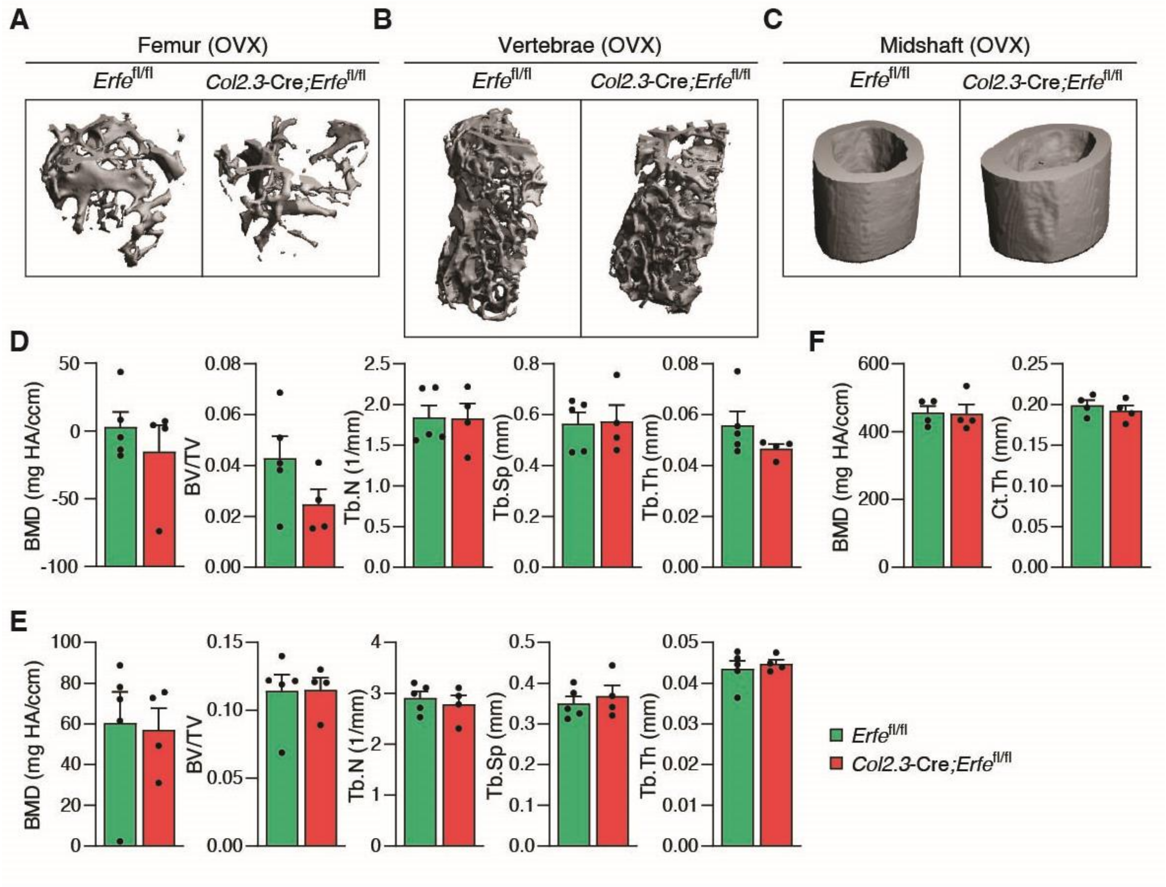

Figure S4: Micro-Architecture Analysis of the Trabecular and Cortical Bone in Ovariectomized Osteoblast-Selective *Erfe* Knockout Mice. (**A**-**C**) Three-dimensional micro-CT images of the femur, lumbar vertebrae, and femoral midshaft of 40-week-old *Erfe*$^{fl/fl}$ and *Col2.3*-Cre; *Erfe*$^{fl/fl}$ female mice following ovariectomy (OVX). (**D-E**) Bone mineral density (BMD), bone volume fraction (BV/TV), trabecular number (Tb.N), trabecular thickness (Tb.Th), and trabecular separation (Tb.Sp) of the femur and lumbar vertebrae from *Erfe*$^{fl/fl}$ and *Col2.3*-Cre; *Erfe*$^{fl/fl}$ mice (*N* = 4-5). (**F**) BMD and cortical thickness (Ct.Th) of femoral midshaft from *Erfe*$^{fl/fl}$ and *Col2.3*-Cre; *Erfe*$^{fl/fl}$ mice (*N* = 4). Data are presented as mean ± SEM.

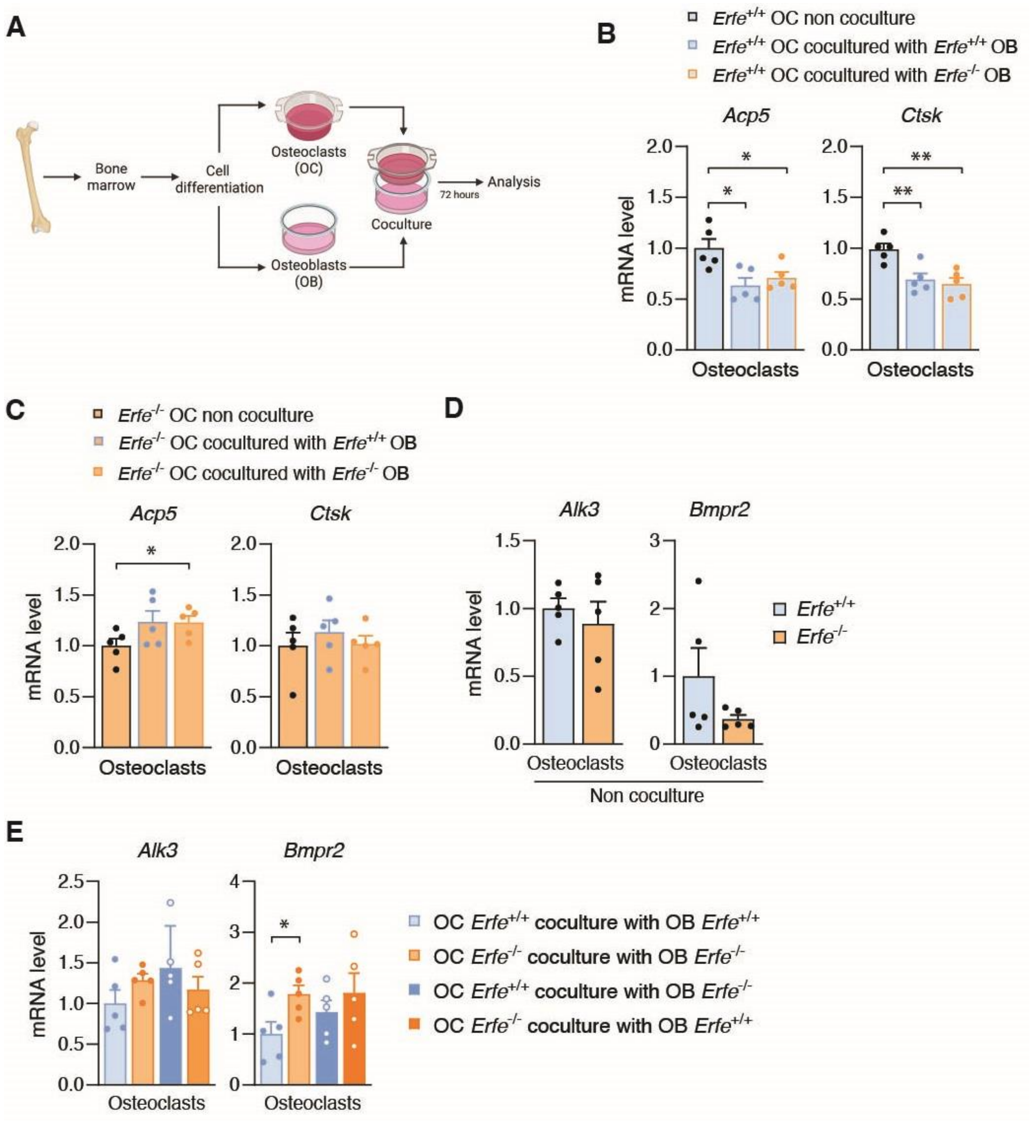

<u>Figure S5</u>: *Erfe* Loss in Osteoblast Promotes Osteoclastogenesis Gene Expression During Osteoblast:Osteoclast Crosstalk in Co-culture. (**A**) Schematic illustrating experimental setup for indirect osteoblast:-osteoclast co-cultures. Mature osteoblasts and osteoclasts were co-cultured with no cell-cell contact for 72 hours using transwell inserts. (**B-C**) *Acp5* and *Ctsk* expression in *Erfe*$^{+/+}$ osteoclasts and *Erfe*$^{-/-}$ osteoclasts under non co-cultured conditions, co-cultured with *Erfe*$^{+/+}$ osteoblasts (OB), and co-cultured with *Erfe*$^{-/-}$ OB (*N* = 5). (**D**) mRNA levels of BMP receptor genes *Alk3* and *Bmpr2* in osteoclasts non coculture *(N* = 5). (**E**) *Alk3* and *Bmpr2* expression of *Erfe*$^{+/+}$ and *Erfe*$^{-/-}$ osteoclasts co-cultured with *Erfe*$^{+/+}$ or *Erfe*$^{-/-}$ osteoblasts (*N* = 5). Data are presented as mean ± SEM. *$P < 0.05$, **$P < 0.01$ using two-tail Student *t* test.

Tables

Table S1: PCR primers for genotyping

Primer name	Sequence	Product size
Primer set 1 *(the 5' loxP site)*	Forward:5'-TATGTGTGTGTGGTCAGCCC-3' Reverse:5'-GGAGGTCCTTAGGGCTCTCA3'	394 bp Floxed *Erfe* 354 bp WT
Primer set 2 *(the 3' loxP site)*	Forward:5'-CCAGGCCCCTTTATCCCATC-3' Reverse:5'-ACAGAAGCAGGAAAGGGCTC-3'	400 bp Floxed *Erfe* 360 bp WT
Universal-cre	Forward:5'-CAAGTGACAGCAATGCTGTTTCAC-3' Reverse:5'-CAGGTATCTCTGACCAGAGTCATC-3'	550 bp
Erfe$^{-/-}$	Forward:5'-GCAGCGCATCGCCTTCTATC-3' Reverse:5'-GACCGTCACTGAGGTTCCAC-3'	390 pb
Erfe$^{+/+}$	Forward:5'-GTCAGCCTTACCTGCCCAG-3' Reverse:5'- GACGTGAATCTCAGTCTGGC-3'	216 pb

Table S2: Oligonucleotide primers for Real-time qPCR

Gene name	Forward (5'−3')	Reverse (5'−3')	Reference
Erfe	ATGGGGCTGGAGAACAGC	TGGCATTGTCCAAGAAGACA	(1)
Acp5	ACCTGTGCTTCCTCCAGGAT	TCTCAGGGTGGGAGTGGG	(2)
Runx2	GTGGCCACTTACCACAGAGC	GTTCTGAGGCGGGACACC	(2)
Alp	ACACCTTGACTGTGGTTACTGCTGA	CCTTGTAGCCAGGCCCGTTA	(2)
Osx	TGAGGAAGAAGCCCATTCAC	GTGGTCGCTTCTGGTAAAGC	(2)
Col1a1	CCTGGCAAAGACGGACTCAAC	GCTGAAGTCATAACCGCCACTG	(2)
Rankl	CAGCCATTTGCACACCTCAC	GTCTGTAGGTACGCTTCCCG	(2)
Opg	ACAGTTTGCCTGGGACCAAA	CAGGCTCTCCATCAAGGCAA	(2)
Bmp2	GCGCAGCTTCCATCACGAAG	ATTGAAGAAGAAGCGCCGGG	This study
Bmp4	TCCGTAGTGCCATTCGGAGC	GCCTCCTAGCAGGACTTGGC	(3)
Bmpr1a	CCCGATTTATGAAAATATGCATCGC	GCAATGACTTTCACCTGCTGCT	This study
Bmpr2	AGCTGCTGCTTCCTAGCTACTAC	ACACTTACCAGGTCTGTGGCTT	This study
Hprt	GCAGTCCCAGCGTCGTGATT	GCCACAATGTGATGGCCTCC	This study
Actin	TTCTTTGCAGCTCCTTCGTT	ATGGAGGGGAATACAGCCC	(2)

SI Materials and Methods

Histomorphometry analysis

Mice received intraperitoneal injection of 15 mg/kg calcein (#C0875, Sigma) at day -8 and 90 mg/kg xylenol (#398187, Sigma) at day -2 prior to sacrifice. Femur and lumbar vertebra were dissected and immediately fixed in 10% neutral buffered formalin. After 24 h, bones were washed under running water for 30 min and immersed in 30% sucrose (#S0389, Sigma) in PBS for 24-72 h at 4°C. Bones were then embedded in a cryomold with OCT (#23-730-571, Fisher Scientific), prior to section at -25°C. The distance between xylenol and calcein lines in the bone sections were measured to yield mineral apposition rate (MAR). Von Kossa staining kit (#CSC0125P, StatLab) was used to measure trabecular bone volume fraction (BV/TV, %) and trabecular thickness (Tb.Th) based on calcium deposits. Sections were visualized under the Zeiss AXIO observer Z1 inverted microscope. Histopathological images were analyzed using QuPath 0.5.

SI References

1. L. Kautz *et al.*, Identification of erythroferrone as an erythroid regulator of iron metabolism. *Nat Genet* **46**, 678–684 (2014).
2. M. Castro-Mollo *et al.*, The hepcidin regulator erythroferrone is a new member of the erythropoiesis-iron-bone circuitry. *eLife* **10**, e68217 (2021).
3. P. Na-Phatthalung *et al.*, Erythroferrone derived from osteoblasts regulates stress erythropoiesis. *Proceedings of the National Academy of Sciences* **123**, e2537627123 (2026).
